# Systematic characterization of the effective constituents and molecular mechanisms of Ardisiae Japonicae Herba using UPLC-Orbitrap Fusion MS and network pharmacology

**DOI:** 10.1371/journal.pone.0269087

**Published:** 2022-06-15

**Authors:** Suxiang Feng, Jie Yuan, Di Zhao, Rongrong Li, Xuefang Liu, Yange Tian, Jiansheng Li

**Affiliations:** 1 Academy of Chinese Medicine Sciences, Henan University of Chinese Medicine, Zhengzhou, Henan, China; 2 Collaborative Innovation Center for Chinese Medicine and Respiratory Diseases co-constructed by Henan province & Education Ministry of P. R. China, Zhengzhou, Henan, China; 3 College of Pharmacy, Henan University of Chinese Medicine, Zhengzhou, Henan, China; 4 The First Affiliated Hospital, Henan University of Chinese Medicine, Zhengzhou, Henan, China; Foshan University, CHINA

## Abstract

**Objective:**

Ardisiae Japonicae Herba (AJH), the dried whole herb of Ardisia japonica (Thunb.) Blume [Primulaceae], has been used in treating chronic obstructive pulmonary disease (COPD) in China. However, the material basis and molecular mechanisms of AJH against COPD remain unclear. Therefore, in this study, we attempt to establish a systematic approach to elucidate the material basis and molecular mechanisms through compound identification, network analysis, molecular docking, and experimental validation.

**Methods:**

Ultra-high performance liquid chromatography-Orbitrap Fusion mass spectrometry (UPLC-Orbitrap Fusion MS) was used to characterize the chemical compounds of AJH. The SwissTargetPrediction, String and Metascape databases were selected for network pharmacology analysis, including target prediction, protein-protein interaction (PPI) network analysis, GO and KEGG pathway enrichment analysis. Cytoscape 3.7.2 software was used to construct a component-target-pathway network to screen out the main active compounds. Autodock Vina software was used to verify the affinity between the key compounds and targets. TNF-α-stimulated A549 cell inflammation model was built to further verify the anti-inflammatory effects of active compounds.

**Results:**

Altogether, 236 compounds were identified in AJH, including 33 flavonoids, 21 Phenylpropanoids, 46 terpenes, 7 quinones, 27 steroids, 71 carboxylic acids and 31 other compounds. Among them, 41 compounds were selected as the key active constituents, which might exhibit therapeutic effects against COPD by modulating 65 corresponding targets primarily involved in inflammation/metabolism/immune-related pathways. The results of molecular docking showed that the key compounds could spontaneously bind to the receptor proteins with a strong binding ability. Finally, the anti-inflammatory effects of the three active compounds were validated with the decreased levels of Interleukin-6 (IL-6) and Matrix Metalloproteinase 9 (MMP9) in TNF-α-induced A549 cells model.

**Conclusion:**

This study clarified that AJH may exert therapeutic actions for COPD via regulating inflammation/immune/metabolism-related pathways using UPLC-Orbitrap Fusion MS technology combined with network pharmacology for the first time. This study had a deeper exploration of the chemical components and pharmacological activities in AJH, which provided a reference for the further study and clinical application of AJH in the treatment of COPD.

## Introduction

Chronic obstructive pulmonary disease (COPD) is a serious chronic inflammatory disease mainly characterized by persistent respiratory symptoms and irreversible airflow restriction caused by significant exposure to harmful gases or particles [1]. COPD is considered as the third most life-threatening disease worldwide [2]. Tobacco smoking, biomass fuel exposure, air pollution and host factors are risk factors for COPD [3–5]. Chronic inflammation, which leads to narrow small airways, structural changes and destruction of lung parenchyma, is the primary cause of COPD [6,7]. At present, the commonly used drugs of COPD are bronchodilators, beta 2-agonists, anti-inflammatory agents (such as glucocorticoids, inhaled corticosteroids, antibiotics etc.) and anti-muscarinic drugs in clinical treatment. However, these drugs have limited aftereffect and many side effect [8–10]. In recent years, Chinese medicine (CM) has been widely used in the treatment of COPD because of its remarkable curative effect of reducing the symptoms of cough, phlegm, and delaying the decline of lung function [11]. Pharmacological studies revealed that Chinese medicine (CM) could be applied to treat COPD by regulating various signaling pathways such as JAK2/STAT3, PI3K/Akt/NF-kappaB and EGFR-PI3K-AKT signal pathways, et, al. [12–18].

Ardisiae Japonicae Herba (AJH) is the whole herb of Ardisia japonica (Thunb.) Blume [Primulaceae], which has anti-inflammatory [19], anti-cancer [20,21], anti-bacterial [22], and anti-viral effects [23,24], has been widely used in clinical treatment of respiratory tract with reliable curative effect, such as chronic bronchitis, pulmonary tuberculosis, upper respiratory tract inflammation and COPD [25–30]. Pharmacodynamic studies clarified that AJH may directly down-regulate the expression of Smad3 and TGF-β1, or inhibit the TGF-β1/Smad3 signaling pathway by increasing the expression of DCN to achieve airway remodeling intervention in COPD rats [31,32]. Moreover, the clinical application of AJH has a history of hundred years and has been recorded in a variety of classical literature [33]. In Bencao Shiyi and Fenlei Caoyao Xing, AJH was thought to have the effects of reducing swelling, resolving phlegm and relieving cough, and commonly used for cough, wheeze, blood in sputum, chronic bronchitis and damp heat jaundice. Many researches have investigated the capacity of AJH for preventing respiratory diseases, and indicated the efficacy of AJH in the prevention of COPD [34–36]. The main chemical components isolated from AJH are flavonoids, coumarins, triterpenes, phenols, polysaccharides and essential oils [37–40]. Of these, flavonoids and phenylpropanoids have the activities of anti-inflammation and anti-oxidation by inhibiting the expression of TNF-α, IL-6 [41–43]. Chang et al. isolated and characterized twenty-one triterpene saponins from AJH [21]. Huang et al. isolated quercetin, Ardisinol Ⅰ and Ardisinol Ⅱ from AJH [44]. In addition, fifteen compounds from AJH were investigated by K.Y. Yu through HPLC-QTOF-MS [45]. However, there are limited research to investigate the systematical material basis and the molecular mechanisms of AJH, more studies are urgently needed to clarify the bioactive components in AJH, and further clarify its medicinal and clinical application values.

**Fig 1:**
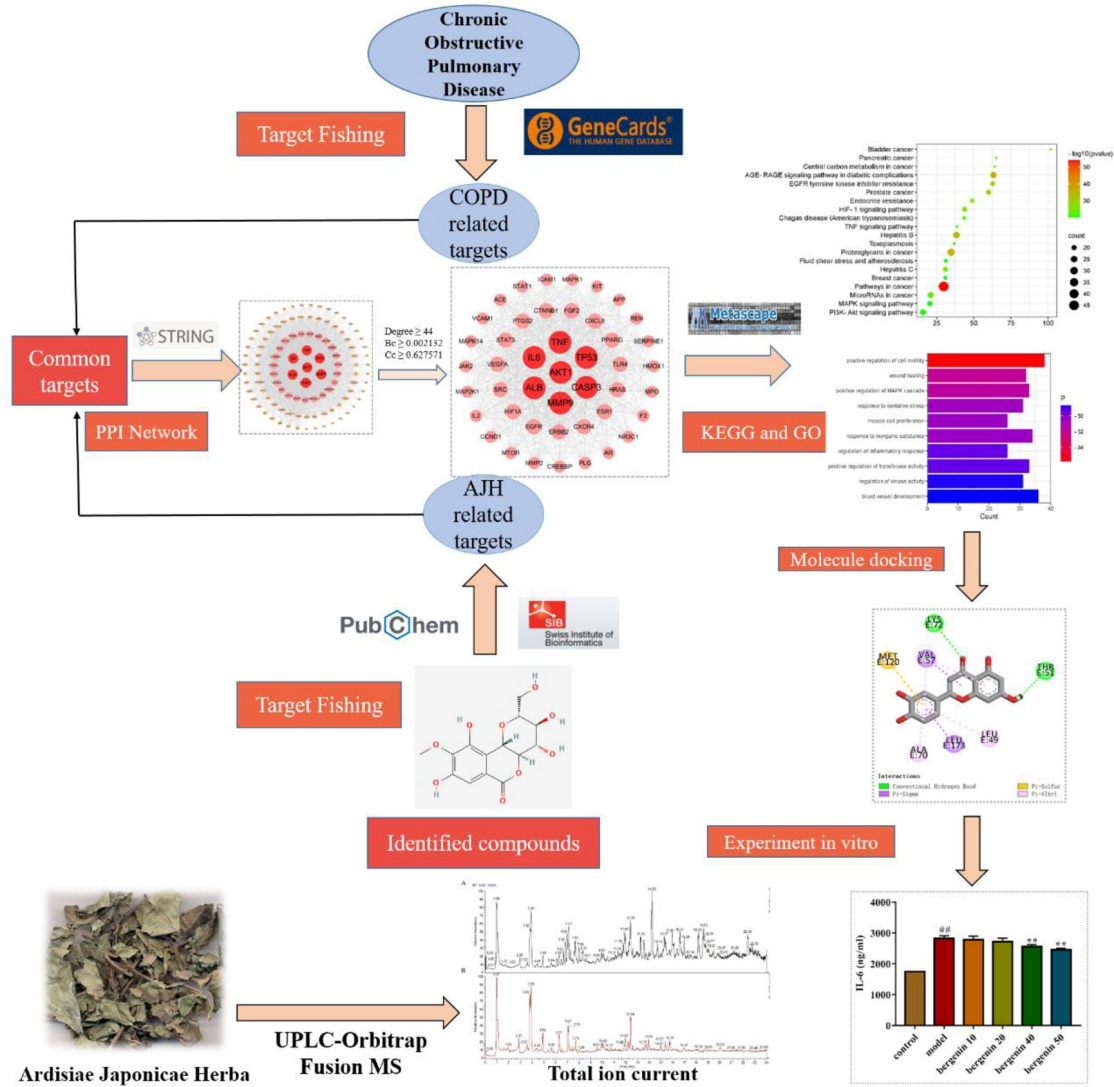
The whole framework of the study.

In this study, we developed a comprehensive method to clarify the material basis and molecular mechanisms of AJH for treating COPD by combining UPLC-Orbitrap Fusion MS analysis and network pharmacology. UPLC-Orbitrap Fusion MS technology was performed to characterize the chemical profile of AJH. Network pharmacology and molecular docking were used to conduct data mining on its chemical components to screen the bioactive components of AJH and predict the underlying mechanisms relevant to COPD treatment, and the effects of potentially bioactive components were further validated in TNF-α-induced A549 cells in vitro. The protocol is shown in Fig 1.

## Materials and methods

### Drugs and reagents

Methanol and acetonitrile (HPLC-grade) were obtained from TEDIA (Fairfield, OH, USA), formic acid (MS-grade) was obtained from Thermo Fisher (Waltham, USA). All other chemicals and reagents were analytical grade. Ultra-pure water was obtained through a Milli-QPOD water purification system (Millipore, Merk, USA). Ardisiae Japonicae Herba pieces were obtained from Jiangxi Zhangshu Tianqitang Traditional Chinese Medicine Co., Ltd (Jiangxi, China, lot no.: 20190804, 20190728, 20190801), and authenticated by Professor Sui-Qing Chen, Henan University of Chinese Medicine, Zhengzhou, China. The voucher specimens were stored at Scientific Research Center, Henan University of Chinese Medicine, Zhengzhou, China.

As reference standards, twenty-nine pure compounds were used. Twenty-six reference standards, including Methyl salicylate (CHB190925), Palmitic acid (CHB181119), Kaempferol (CHB190127), Embelin (CHB190829), Hydroxygenkwanin (CHB180611), Rapanone (CHB190723), Epigallocatechin gallate (CHB180307), Myricitrin (CHB180611), Isoeugenol (CHB190830), Gallic acid (CHB180114), Ethyl gallate (CHB180116), Caryophyllene oxide (CHB180624), Myristic acid (CHB190102), Taxifolin (CHB190105), Myricetin (CHB180614), Astilbin (CHB190107), Scopoletin (CHB180210), Fraxetin (CHB180112), Cardamomin (CHB180312), Eriodictyol (CHB190123), Cianidanol (CHB180809), Gallocatechin (CHB200915), Arctigenin (CHB180131), Epicatechin gallate (CHB180305), Rutin (CHB190110), Nictoflorin (CHB190102) were obtained from Chengdu Cloma Biological Technology Co., LTD (Chengdu, China); Quercetin (MUST-20101104) and Luteolin (MUST-14050411) were obtained from Chengdu Must Biological Technology Co., Ltd. (Chengdu, China); Bergenin (111532–201604) was obtained from China Food and Drug Testing Institute (Beijing, China), and the purity of each compound was ≥ 98% by HPLC analysis.

Roswell Park Memorial Institute (RPMI) 1640 medium, Radio Immunoprecipitation Assay (RIPA) Lysis Buffer, Thiazolyl Blue Tetrazolium Bromide (MTT) and trypsin-EDTA were obtained from Beijing solarbio science & technology co., ltd (Beijing, China), Fetal bovine serum (FBS) was purchased from Zhejiang Tianhang Biotechnology Co., Ltd. (Zhejiang, China), the ELISA kits for IL-6 and MMP9 (the sensitivity was < 0.3 pg/mL and 5 pg/mL, respectively) were obtained from BOSTER Biological Technology co., ltd (Wuhan, China).

### Qualitative analysis of AJH

#### Sample preparation

AJH powder (1.0 g) was accurately weighed and added in a 150 mL round-bottom flask with 100 mL methanol, mixed well, reflux-heated for 1h twice and filtered. Obtained filtrates were combined and dried by centrifugation. The residue was redissolved with methanol (HPLC-grade) and diluted to 10 mL volumetric flask. The solution was filtered using 0.22 μm microporous membrane and stored at 4°C before analysis.

#### Standard solution preparation

Accurately weigh an appropriate amount of each reference standard into a 10 mL volumetric flask, add methanol to dissolve and dilute to the mark, single-component reference stock solutions were obtained and stored at 4°C before use. Then, proper amounts of reserve solutions were added to a 25 mL volumetric flask, methanol (HPLC-grade) was added to reach scale, the mixed reference solution was obtained. The solutions were filtered through 0.22 μm microporous membranes and stored at 4°C before use.

#### Chromatographic and mass spectrometric conditions

Chemical identification was conducted on an ultra-performance liquid chromatography-orbitrap fusion mass (UPLC-Orbitrap Fusion MS) (Thermo Fisher, Waltham, USA) equipped with an electrospray ionization source (ESI). Thermo Scientific Accucore^TM^C_18_ column (2.1 mm × 100 mm, 2.6 μm) was applied at a constant flow rate of 0.2 mL/min at 30°C. The mobile phase was made up of methanol (A) and 0.1% formic acid aqueous solution (B), and the gradient elution was as follows: 0~2 min (93~75% B), 2~10 min (75~20% B), 10~15 min (16~12% B), 15~20 min (12~10% B), 22~24 min (10~0% B). The injection volume was 5 μL.

The acquisition parameters of orbitrap fusion were set as follows: Vaporizer temperature: 275°C; Ion Transfer Tube temperature: 300°C; Carrier gas (N2); Sheath gas and Aux gas flow were set as 35 arb and 5 arb, respectively; spray voltage were set at 3.5 kV, 2.8 kV (positive and negative ion mode, respectively); collision energy was 35–55 eV. The sample was analyzed in both positive and negative ions Full MS/dd-MS2 modes with the first-level full scan (resolution: 120,000) and the second-level scan (resolution: 60,000), and the mass range was recorded from m/z 100–1200.

#### Data processing

The chemical compounds of AJH were collected from existing databases, including the Traditional Chinese Medicine Systems Pharmacology Database and the Analysis Platform (TCMSP, http://lsp.nwu.edu.cn/tcmsp.php), Traditional Chinese medicine integrative database (TCMID, http://47.100.169.139:8000/tcmid/) and SciFinder (https://scifinder.cas.org/) database. Then, a database of AJH ingredients was established. AJH was identified by the optimized UPLC-Orbitrap Fusion MS method. The possible chemical composition (with an error of less than 5 ppm) was determined using Xcalibur based on map data, precise molecular weight and resulting fragment ions. The data was analyzed using Compound Discoverer software to integrate ion peak information, attribution information, ChemSpider, mzCloud and other databases of characteristic fragments integrated with existing chemical composition information reports. The structure of compounds was inferred using the cracking prediction of MassFrontier and its cracking rule.

### Network pharmacology

#### Target prediction

The molecular structures of components identified in AJH were downloaded from PubChem database (https://pubchem.ncbi.nlm.nih.gov/) and saved in SDF format. These SDF documents were imported to SwissTargetPrediction database (http://www.swisstargetprediction.ch/) for target prediction. For more accurate prediction, relevant parameters were set (probability ≥ 0.1). In addition, the COPD-related targets were selected from GeneCards database (https://www.genecards.org/) by using “Chronic Obstructive Pulmonary Disease” as the keywords. Then, the overlapping of AJH prediction targets and COPD-related targets was selected as potential targets.

#### PPI network construction

The PPI network construction and analysis were performed on Cytoscape 3.7.2 software. Potential targets screened above were added to STRING database. The screening condition used was “Homo sapiens,” and the other parameters were set by default, a protein-protein interaction (PPI) network was built. Cytoscape 3.7.2 was used for topological analysis of PPI network to realize its visualization. Using three topological parameters, betweenness centrality (Bc), closeness centrality (Cc) and degree, a topology analysis of the PPI network was performed to determine hub genes for further analysis.

#### Gene Ontology (GO) and Kyoto Encyclopedia of Genes and Genomes (KEGG) enrichment analysis

Potential targets identified above were uploaded into Metascape database (https://metascape.org/gp/index.html) for GO and KEGG analysis to obtain the information of pathways. Bioinformatics Data analysis and Visualization online platform (http://www.bio-informatics.com.cn/) was used to conduct GO and KEGG pathway analysis and visualization. In the process, the organism was set as “Homo sapiens”, and significance level was p Ho0.01.

#### Construction of component-target-pathway network

To further clarify the relationship among components, targets and pathways, Cytoscape 3.7.2 software was used to build a component-target-pathway network. The core compound nodes were obtained based on the three parameters: degree, Bc and Cc.

#### Molecular docking

The affinity between the core compounds and targets was verified by Autodock Vina software The 3D structures of core targets in the first five degrees of AJH in COPD treatment were obtained from the RCSB PDB database (https://www.rcsb.org/). Pymol 1.8 software was used to remove water molecules and separate the primary ligand. After saving, the structure was imported into Autodock Tools 1.5.6 and saved in “pdbqt” format. Chem 3D software was used to download the mol2 files of the top 10 core compounds, and they were imported into Autodock Tools 1.5.6 and saved in pdbqt format. Finally, docking was carried out through Autodock vina 1.1.2. Discovery Studio 4.5 Client was used to visualize docking results and establish a docking interaction model diagram.

### Experimental validation in vitro

#### Cell culture

A549 cells were from Cell Resource Center of Shanghai Academy of Biological Sciences, Chinese Academy of Sciences (Shanghai, China). Cells were cultured in RPMI 1640 complete medium (containing 10% FBS) and kept in an incubator at 37°C and 5% CO2. When the cells were overgrown, they were digested with 0.25% trypsin-EDTA, collected, subcultured or tested.

#### Cell viability assay

A549 cells (1 × 10^4^ cells/well) were planted into 96-well plates (n = 6), and cultured at 37°C and 5% CO2 for 24 hours. Then the cells were treated with PBS or different dosages of bergenin (10, 20, 40, 50 μg/mL), luteolin (0.1, 0.2, 0.4, 0.8 μg/mL) and kaempferol (2.5, 5, 10, 20 μg/mL). After incubation for 24 h, added 10 μl MTT (0.5 mg/mL) to each well, and cultured for 4h. Using DMSO (100 μL/well) to replace cell culture. The absorbance was measured at 570 nm using a microplate reader.

#### IL-6, MMP9 expression

A549 cells (2×10^6^ cells/plate) were incubated in 6-well plates (n = 6) for 24 h, incubated with TNF-α (10ng/mL) for 24 h. Then treated with bergenin (10, 20, 40, 50 μg/mL), luteolin (0.1, 0.2, 0.4, 0.8 μg/mL), and kaempferol (2.5, 5, 10, 20 μg/mL), and incubated at 37°C with 5% CO2 in a humidified atmosphere for 48 h. Supernatant was collected, centrifuged and stored at -80°C for testing. RIPA Lysis Buffer 300 μL/well was added to lysate cells. Ten minutes later, the lysate was collected, the supernatant was collected by centrifugation, and levels of IL-6, MMP9 in the supernatant were determined according to the instructions of ELISA kits.

#### Statistical analysis

IBM SPASS Statistic 26 software was applied for statistical analysis, and data was expressed as mean ± standard deviation (SD). Analysis of variance (ANOVA) was used to analyze the differences between multiple groups. P-values < 0.05 was considered to be statistically significant.

## Results

### Characterization and identification of components in AJH

**Fig 2:**
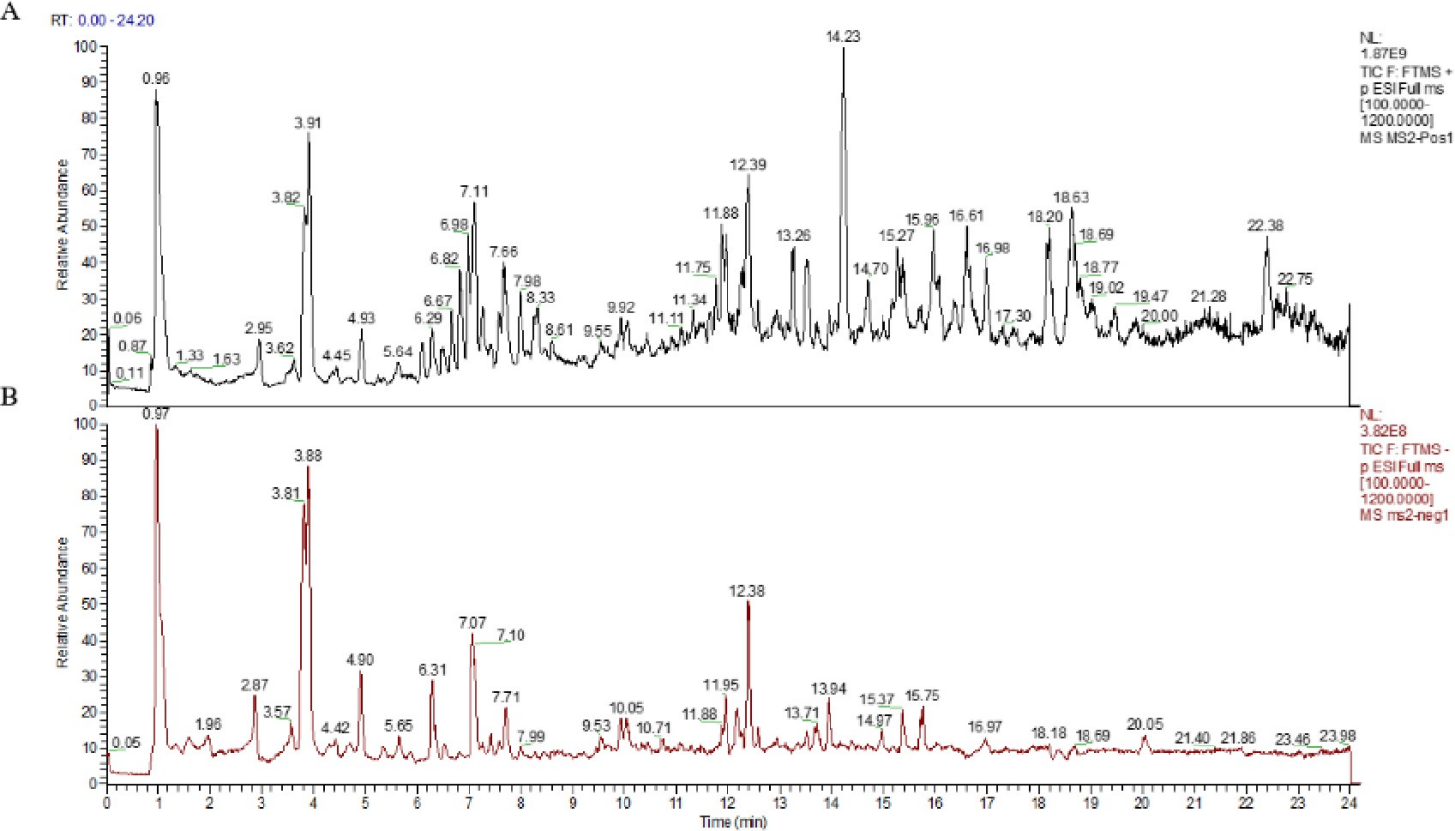
The total ion chromatograms (TICs) of AJH (A. positive ion mode. B. negative ion mode).

Total ion chromatograms (TICs) of AJH are presented in Fig 2 (TICs of the three batches of AJH are as shown in S1 Fig), 236 chemical compounds (162 in ESI^+^ and 74 in ESI^-^) were identified in AJH, including 33 flavonoids (F), 21 Phenylpropanoids (P), 46 terpenes (T), 7 quinones (Q), 27 steroids (S), 71 carboxylic acids (C) and 31 others (O) based on the reference standards, ingredients database, mass fragment mode and mass spectrum library with UPLC-Orbitrap Fusion MS system. Among them, 29 compounds (F4, F6, F8, F10, F13-14, F16, F19, F22-23, F25-28, F31, P4, P7, P13, P20, T15, T26, Q4, Q6, C5, C16, C20, O19-20 and O22) were identified by comparing with the reference standards. The details of 236 components, including compounds name, formula, retention time (tR), and fragments ions are presented in **S1 Table**.

### Network pharmacology analysis

#### Potential targets prediction

**Fig 3:**
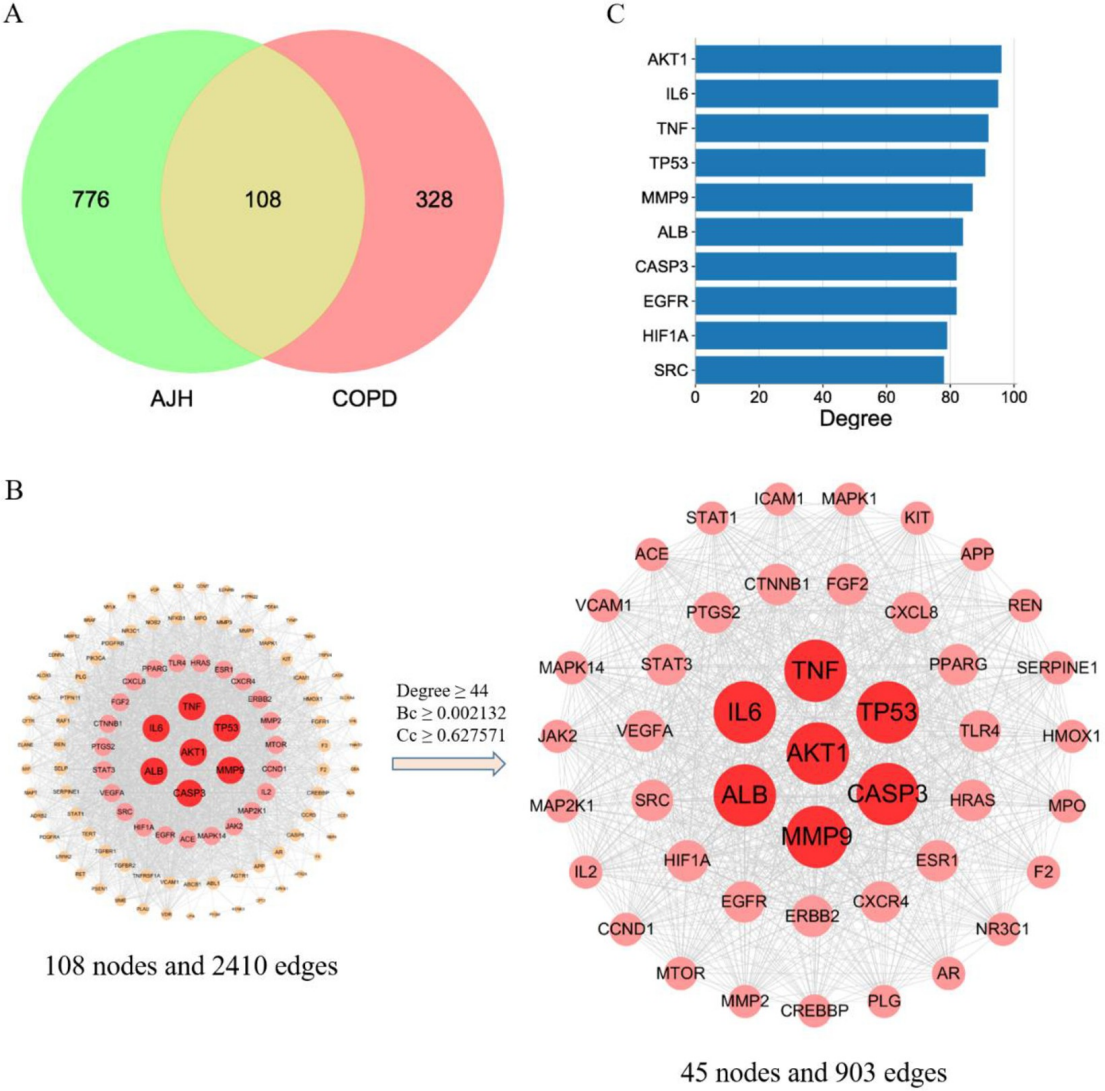
Network of common targets. (A) Intersection of Venn diagram. (B) PPI network of common targets. (C) Targets in the top ten degree.

After removing repeated targets, 884 targets were collected based on 236 primarily identified compounds using the SwissTargetPrediction database (**S2 Table**). Only 207 compounds had relevant targets when the probability ≥ 0.1. In addition, 6412 COPD-related genes were collected from the GeneCards database. To ensure the precision of target collection, based on the parameter of “Relevance score,” the index above the third median value was selected as the key index, and 436 COPD-related targets were obtained (**S2 Table**). After taking the intersection of 884 predicted targets and 436 COPD-related targets, 108 common targets were obtained which means that they could be the main potential targets of AJH in COPD treatment (Fig **3**A)

#### Construction and analysis of the PPI network

After inputting these common targets into STRING database, we obtained a PPI network comprising 108 nodes and 2410 edges (Fig **3**B). Using the three parameters degree, Bc and Cc, the index above the median value was selected as the key index, the threshold value of screening was degree ≥ 44, Bc cnd Cc, the index above the median value was selected as the **Fig 3**). In addition, genes in the top ten degree were selected (**Fig 3C**), the results showed that AKT Serine/Threonine Kinase 1 (AKT1), Interleukin-6 (IL6), Tumor necrosis factor (TNF), Tumor Protein P53 (TP53) and Matrix Metalloproteinase 9 (MMP9) were the most vital targets of PPI, which may be the main targets of AJH in COPD treatment.

#### GO and KEGG pathway enrichment analysis

**Fig 4:**
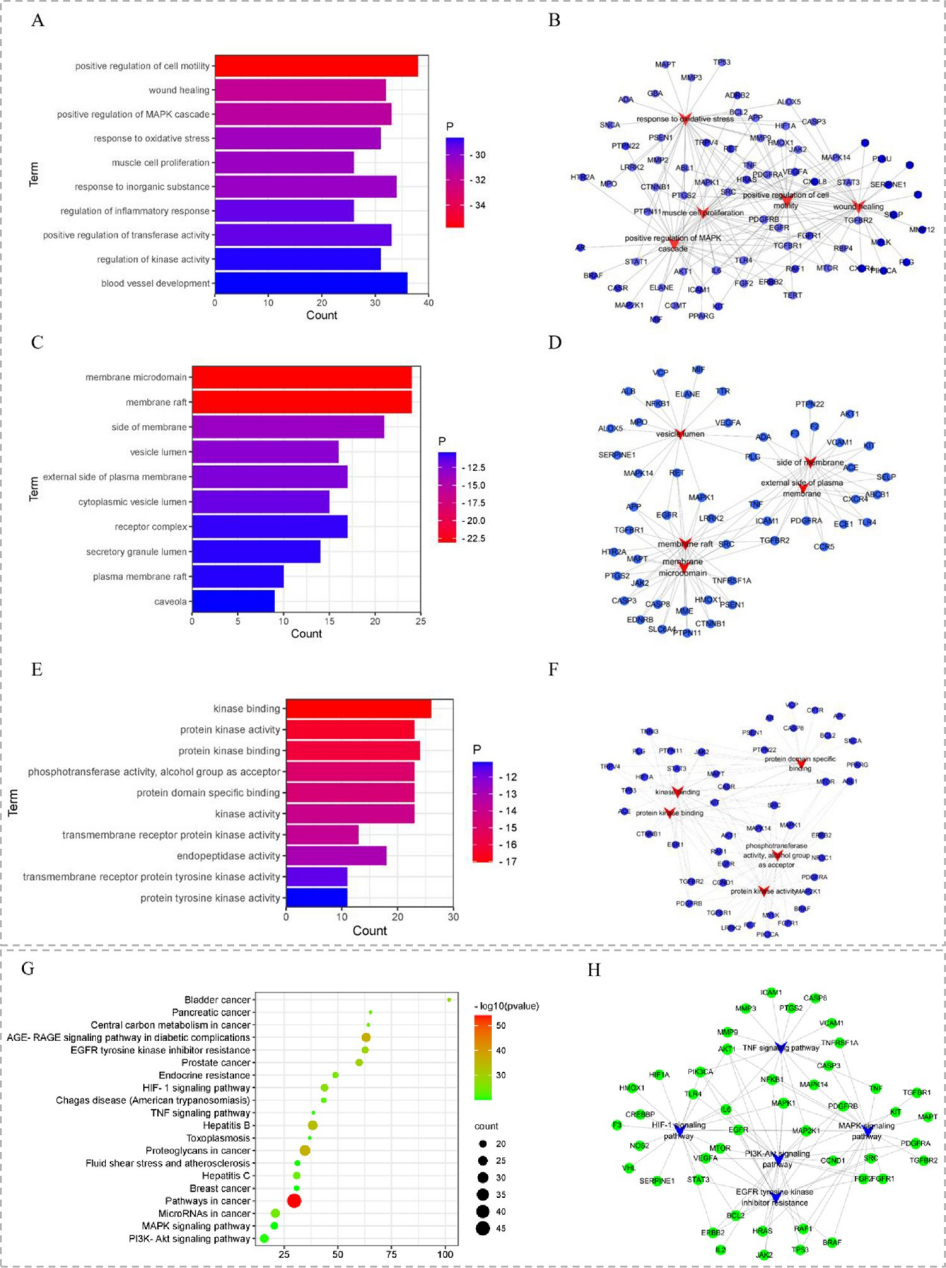
GO and KEGG enrichment of potential targets. (A, C, E: Top 10 significantly enriched terms in BP, CC and MF, respectively. B, D, F: The top five terms and corresponding targets in BP, CC and MF, respectively). (G) Top 20 pathways enriched. (H) Pathway-target network (the five COPD-related pathway and corresponding targets).

GO and KEGG enrichment analysis on the 108 potential targets was conducted through Metascape database to gain insight into the mechanisms of AJH in treating COPD. Accordingly, 2, 297 GO terms and 167 KEGG pathways with P-value valuerdingly, 2, 297 (**S2 Table**). Moreover, the term/pathway-target networks were constructed (as shown in Fig 4).

Gene ontology enrichment analysis consisted of three parts: biological process (BP), cellular component (CC) and molecular function (MF). The top 10 important terms in BP, CC and MF are shown in Fig 4A–4F. The enrichment results showed that 2, 063 enrichment terms are related to BP, which mainly cover positive regulation of cell motility, positive regulation of MAPK cascade, response to oxidative stress and regulation of inflammatory response. In addition, 134 enrichment results were related to MF, including kinase binding, protein kinase activity and phosphotransferase activity. 100 CC items were also collected, of which membrane raft, membrane microdomain and side of membrane were the most enriched. The top 20 KEGG pathways enriched were shown in Fig 4G and 4H. Among them, the pathways significantly related to COPD treatment include EGFR tyrosine kinase inhibitor resistance, HIF-1, TNF, PI3K-Akt and MAPK signaling pathway, etc.

#### Component-target-pathway network

**Fig 5:**
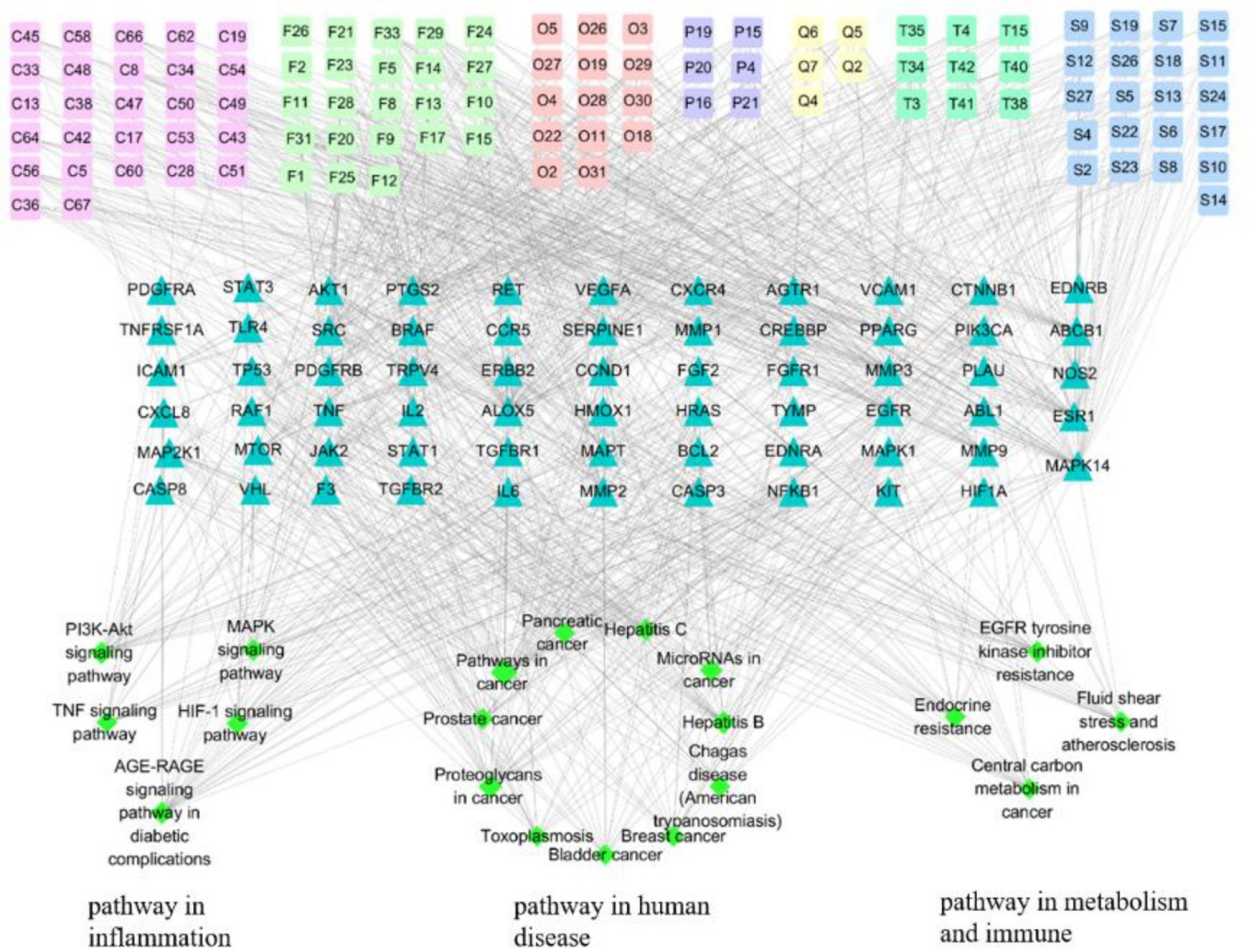
Component-target-pathway network comprised 190 nodes and 1042 edges. The rectangle nodes represent 105 components identified in AJH, the triangle nodes represent 65 COPD-related targets, and the diamond nodes represent the top 20 enriched pathways. The edges represent interactions between compounds, targets and pathways.

The component-target-pathway network (Fig 5) was constructed to clarify the contact among components, key targets and pathway, which comprised 190 nodes and 1042 edges. The rectangle nodes represent 105 components identified in AJH, the triangle nodes represent 65 COPD-related targets, and the diamond nodes represent the top 20 enriched pathways. The edges represent interactions between compounds, targets and pathways. According to the topological parameters, 41 hub components were obtained, including 12 flavonoids, 11 steroids, 7 carboxylic acids, 6 terpenes, 2 phenylpropanoids, 1 quinone, 1 ketone and 1 phenol. The detailed information of components with degree values in the top twenty are shown in Table 1. Among them, Hydroxygenkwanin, luteolin, kaempferol, Idebenone, Myricetin 3-O-galactoside, Linoleic acid, Alpinetin, Bergenin and Digitoxigenin showed strong interactions with 10 or more COPD targets, and might be the potential bioactive constituents of AJH against COPD.

**Table 1:** Topological properties of hub components.

ID	components	Bc	Cc	Degree
F31	Hydroxygenkwanin	0.03934312	0.43661972	15
F25	Luteolin	0.03669989	0.43661972	14
F28	Kaempferol	0.01581494	0.4025974	11
Q5	Idebenone	0.01506443	0.34521158	11
F33	(-) Alpinetin	0.02813808	0.38847118	10
C45	Linoleic acid	0.02027674	0.4200542	10
F12	Myricetin 3-O-galactoside	0.01617418	0.37170264	10
P4	Bergenin	0.01301043	0.39641944	10
S7	Digitoxigenin	0.02640662	0.42234332	10
F24	Laricitrin	0.00713272	0.34521158	9
S8	(3beta,5beta,12beta)-3,12,14-Trihydroxycardanolide	0.02181039	0.4200542	9
C8	Mycophenolic acid	0.021414	0.35632184	9
C66	Bis(2-ethylhexyl) phthalate	0.01380206	0.38271605	9
C56	Farnesyl acetate	0.02283748	0.37897311	9
F29	Naringenin	0.02506296	0.37897311	9
O29	2-Methylcardol monoene	0.00942	0.388471	9
T4	Helenalin	0.024118	0.3875	9
S12	Alfaxalone	0.014573	0.388471	9
S4	Cortisol	0.015075	0.388471	9
T34	18-β-Glycyrrhetinic acid	0.014072	0.41779	8

#### Validation by molecular docking

**Fig 6:**
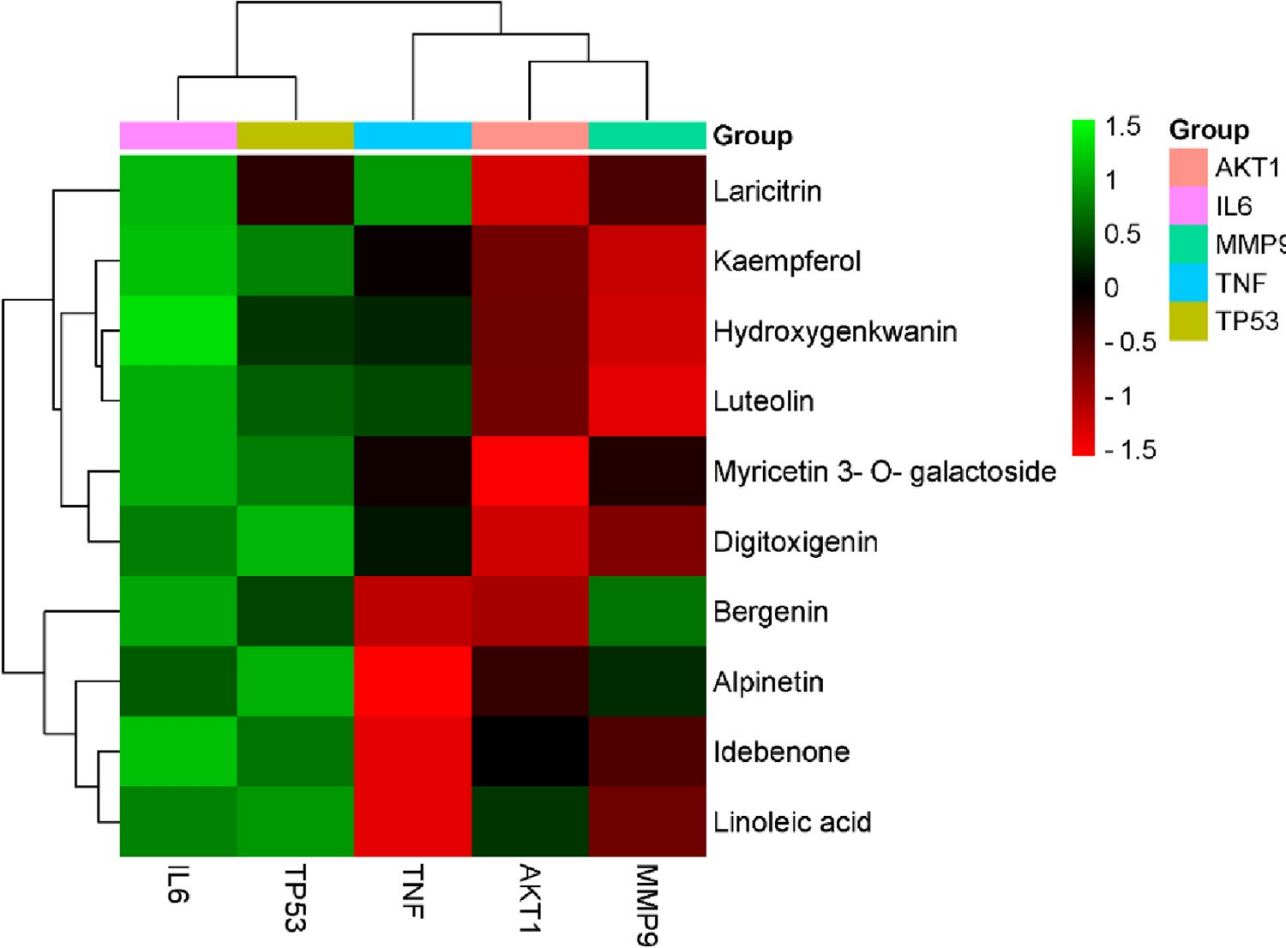
The binding energy between the core compounds with core targets in AJH.

The top ten core compounds were docking with the corresponding five key targets, and the results were analyzed. The basic information about ligands and proteins is shown in Table 2. The results showed that the binding free energy (ΔG in kcal/mol) of compounds for binding to key targets was negative (as shown in **S2 Table**), indicating the ligands molecules could bind to receptor proteins spontaneously. In addition, the binding energy was smaller than -5.0 kJ/mol, which further proved the strong binding energy. The heat map revealed the difference of binding energy between different compounds and targets, as shown in Fig 6.

**Table 2:** The basic information of ligand and protein.

Targets	PDB ID	Ligand ID	Ligand Name
AKT1	2F7Z	6EA	(1S)-1-(1H-INDOL-3-YLMETHYL)-2-(2-PYRIDIN-4-YL- [1,7] NAPHTYRIDIN-5-YLOXY)-EHYLAMINE
IL6	5FUC	NAG	2-acetamido-2-deoxy-beta-D-glucopyranose
TNF	6OP0	A7A	(R)- {1- [(2,5- dimethyl phenyl) methyl] -6-(1-methyl-1H-pyrazol-4-yl)-1H-benzimidazol-2-yl} (pyridin-4-yl) methanol
TP53	5O1C	9GZ	5-(4-fluorophenyl)-3-iodanyl-2-oxidanyl-4-pyrrol-1-yl-benzoic acid
MMP9	6ESM	B9Z	(2~{S})-2- [2- [4-(4- methoxyphenyl) phenyl] sulfanylphenyl] pentanedioic acid

Luteolin has the best binding ability with five core targets. Visualization of the docking results was performed using Discovery Studio 4.5 Client software. The docking of the luteolin, kaempferol and bergenin with target proteins was primarily through thirteen forces, including conventional hydrogen bond, unfavorable donor-donor, unfavorable acceptor- acceptor, unfavorable bump, carbon hydrogen bond, amide-pi stacked, pi-donor hydrogen bond, pi-sigma, pi-pi stacked, pi-sulfur, sulfur-x, pi-alkyl, van der waals (S2 Fig). Luteolin has the strongest binding to MMP9 through 6 forces, mainly including conventional hydrogen bond, which formed hydrogen bonds with MET247 and ARG249 residues. Kaempferol has the strongest binding to MMP9 through 5 forces, which formed hydrogen bonds with ALA189, GLN227, LEU188 and VAI223 residues. Bergenin has the strongest binding to TNF through 4 forces, which formed hydrogen bonds with SER99, HIS73 and residues. These results clarified that the active ingredients had better binding properties to protein targets.

### Experimental validation

According to the network pharmacology, molecular docking analysis and references, three bioactive components including luteolin, kaempferol, bergenin had the better molecular docking results. To further evaluate the results obtained by systematic pharmacologic analysis, these components were selected from AJH to examine the potential effects of anti-inflammatory by using TNF-α-stimulated A549 cells. We employed ELISA assay for IL-6, MMP9 to confirm the predicted anti-inflammatory effects of three bioactive components. Morever, MTT assay and cytokine expression assay were performed at concentrations that did not affect cell activity by comparing references and conducting preliminary experiments.

**Fig 7:**
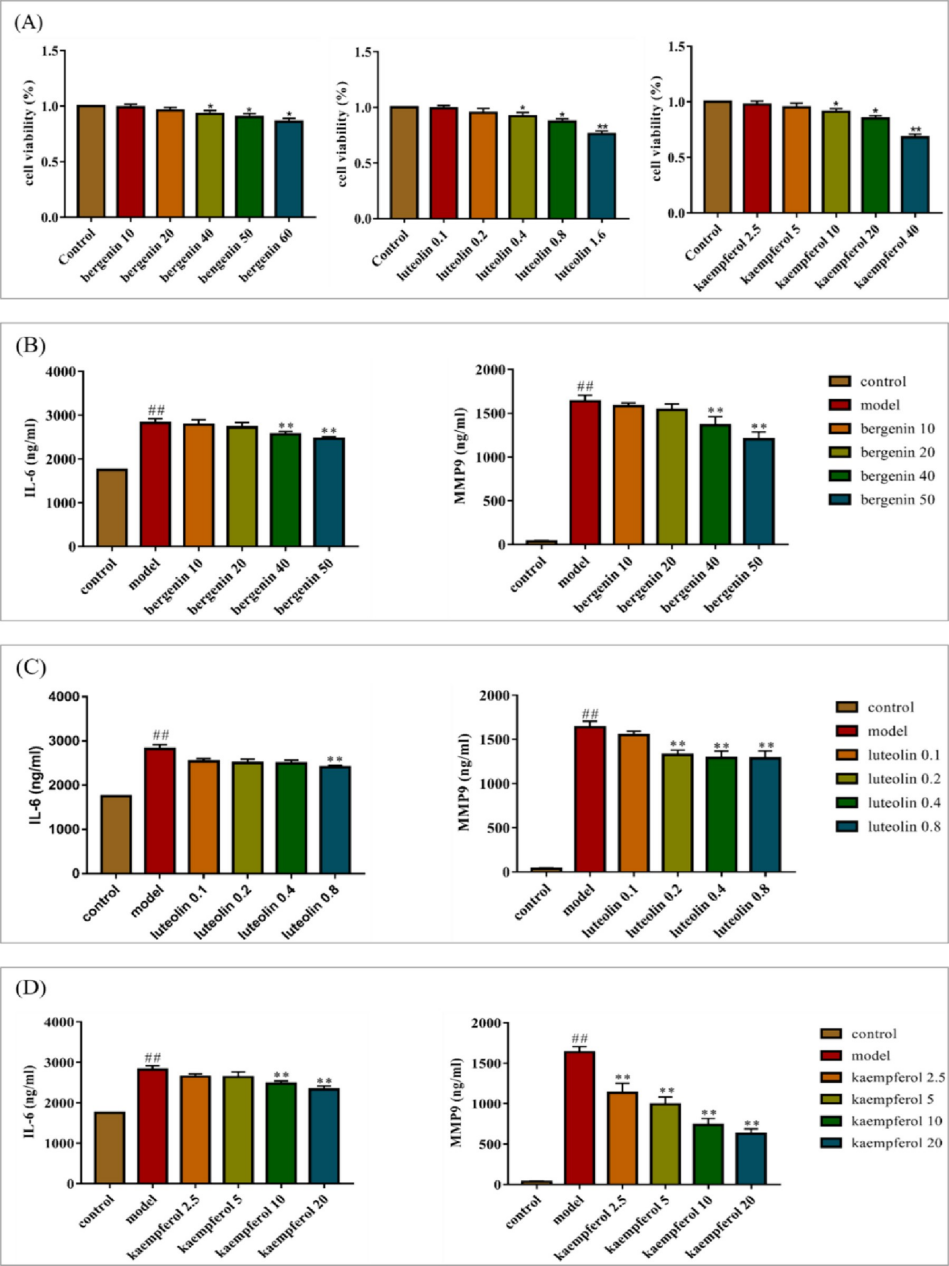
Effect of bergenin, luteolin, kaempferol on A549 cells. A: The effect of bergenin, luteolin, kaempferol on A549 cell viability were detected by MTT analysis. B-D: The effects of different concentrations of bergenin, luteolin, kaempferol on the contents of IL6, MMP9 in COPD model were determined by ELISA, respectively. Values represented as mean ± SD, n = 6. ^##^P < 0.01 versus control group. **P < 0.01, *P < 0.05, versus model group.

The MTT kits were used to assess the cytotoxicity to select proper concentrations of bergenin, luteolin and kaempferol. As shown in Fig 7A, bergenin, luteolin, kaempferol showed cytotoxicity to A549 cells at 60, 1.6 and 40 μg/mL, respectively. Therefore, bergenin (10, 20, 40, 50 μg/mL), luteolin (0.1, 0.2, 0.4, 0.8 μg/mL), kaempferol (2.5, 5, 10, 20 μg/mL) were selected for subsequent experiments. Compared with controls, the contents of IL6 and MMP9 in A549 cells were significantly increased (p<0.01, Fig 7) after TNF-α induction. Compared with the model group, the content of IL6 and MMP9 were decreased in varying degrees after the treatment of three compounds with different concentrations. In A549 cells co-cultured with TNF-α and three compounds, the expression levels of IL-6 and MMP9 reduced significantly (p<0.01) in a dose-reliant pattern. Bergenin with low dose (10 μg/mL) has no anti-inflammatory effects (Fig 7B–7D).

## Discussion

In this study, the chemical components of AJH were identified through a comprehensive approach, including rapid analysis of chemical components, network pharmacology analysis and experiment in vitro, and the molecular mechanisms of its anti-COPD against COPD was discussed. Firstly, the total ion chromatograms of AJH were obtained by UPLC-Orbitrap Fusion MS. Different mobile phase systems and elution gradients were investigated to optimize reliable and effective chromatography conditions. It was found that the elution effect of methanol was better than that of acetonitrile, and the separation effect of each chromatographic peak was better. Adding 0.1% formic acid can improve the peak shape and the mass spectrum response, so we used methanol-0.1% formic acid water as the mobile phase. In addition, due to the different response modes of various compounds in AJH, positive and negative ion-scanning modes were selected for simultaneous detection line monitoring. By using the Compound Discoverer 3.0 software to match the original MS raw data to mzCloud, Chemspider and other built-in databases, a total of 236 chemical constituents (162 in ESI+ and 74 in ESI-) were clarified or preliminarily characterized, mainly including flavonoids, phenylpropanoids and terpenes, which is consistent with the literature [46]. Previous study had shown fifteen compounds from AJH were investigated by K.Y. Yu through HPLC-QTOF-MS [45]. Compared with previous studies on the chemical constituents of AJH, this study identified and compared the chemical constituents of AJH in vitro more comprehensively and systematically. It could be seen that the established UPLC-Orbitrap Fusion MS method has more sensitivity to identify the chemical components in AJH, and the results provided more chemical material basis information for further study of AJH.

Network pharmacology can reflect the contact among components/targets and pathways, and the molecular mechanisms of drug therapy can be clarified by constructing a “component-target-pathway” network. In the present study, 111 of the 236 components characterized of AJH were thought to have potentially critical anti-COPD effects through acting on 108 related targets. To further explore the key pharmaceutically bioactive compounds and underlying mechanisms of AJH against COPD, a multidimensional component-target-pathway network was further constructed and visualized using Cytoscape v3.7.2. Based on three topological parameters, forty-one key active components were screened out, including twelve flavonoids, eleven steroids, seven carboxylic acids, six terpenes, two phenylpropanoids, one quinone and two other compounds, which proved that AJH had a multicomponent synergistic effect in the treatment of COPD. Of these, flavonoids have the activities of anti-inflammation and anti-oxidation by inhibiting the expression of TNF-α, IL-6 and COX-2, which may have greater potential effects on COPD [47–49]. It has been reported that luteolin and kaempferol exert therapeutic effects on COPD by inhibiting lung inflammation through reducing the release of inflammatory cytokines such as IL-6, IL-12 and TNF-α [50,51]. Alpinetin play a protective role in COPD by decreasing the activities of TGF-β1, TNF-α, and α-SMA, inhibiting apoptosis and reducing the release of inflammatory cytokines [52]. Hydroxygenkwanin showed antitumor activity on Non-Small Cell Lung Cancer cells by promoting the degradation of EGFR [53]. Laricitrin is an efficacious immunoadjuvant with great potential to improve the inhibitory effects of lung cancer on differentiation, maturation and function of dendritic cells [54]. In addition, idebenone, linoleic acid, bergenin and digitoxigenin also have relatively high degree values. Among them, bergenin, the main phenylpropanoids in AJH, can significantly improve lung damage and reduce the levels of inflammatory factors such as TNF-α, IL-6, IL-1β and PEG2, and can significantly improve lung function and blood gas indexes in COPD model rats [55,56]. The mechanisms of these active ingredients cover multi-aspects of the pathogenesis of COPD, and the targets/pathways obtained are basically consistent with our predicted results, indicating that the comprehensive pharmacological strategy has certain predictive accuracy.

The results of network pharmacology showed that these active components may effectively improve the pathologic status of COPD patients by modulating sixty-five corresponding targets primarily involved in inflammation and oxidation related pathways. Among the predicted potential targets, AKT1, IL6, TNF, TP53, MMP9, CASP3 and ALB had high frequency, and they were involved in several biological processes, including positive regulation of MAPK cascade, positive regulation of cell motility, response to oxidative stress, regulation of inflammatory response, which were closely related to the pathogenesis of COPD. KEGG enrichment analysis showed that the potential targets were highly related to human disease for cancer, infectious diseases, metabolic diseases, immune system and signal transduction, including the pathways in cancer, AGE-RAGE signaling pathway in diabetic complications, endocrine resistance, EGFR tyrosine kinase inhibitor resistance, HIF-1, TNF, MAPK and PI3K-Akt signaling pathway, etc. Combined with the COPD pathogenesis and common signaling pathways, it is inferred that AJH exerted therapeutic effects on COPD through the following aspects: anti-inflammatory, antioxidant and immune regulation. TNF-α is one of the key signal molecules of high secretion of airway mucus, and it is also a biological indication of COPD grade and curative efficacy [57]. TNF-α can activate neutrophils and macrophages to release more cytokines such as IL-6 and IL-8, thus speeding up the inflammatory response process [58]. Abnormal activation of TNF signaling pathway plays a key role in goblet cell metaplasia and airway mucus hypersecretion [59]. The binding of advanced glycation end-product receptor (RAGE) to AGEs (glycosylated and oxidized proteins, lipids or nucleic acids) can induce pro-inflammatory response, and the AGE-RAGE pathway has become a significant signal transduction pathway, affecting immune and oxidative stress responses by activating MAPK and NF-κB pathways [60,61]. Thus, we speculated that AJH might play an anti-inflammatory and antioxidant role in the treatment of COPD by regulating TNF, MAPK signaling pathway and AGE-RAGE signaling pathway in diabetic complications. In addition, the enrichment results showed that AJH could indirectly participate in the treatment of COPD through central carbon metabolism and endocrine resistance.

We selected molecular docking to validate the binding of core compounds and targets related to inflammation and oxidative stress. The binding free energy of compounds to the key targets was smaller than -5.0 kJ/mol, which indicated that the ligand molecules could spontaneously bind to receptor proteins with strong binding ability. Studies have shown that alveolar epithelial cells A549 are one of the mediators for COPD detection [62,63]. Inflammation is the most important cause of COPD [6]. Based on our previous research, TNF-α can inhibit the growth of A549 cells, induce the secretion of inflammatory factors such as IL8 and MMP-9, and activate NF-κB and MAPK signaling pathway, which are consistent with the pathological development mechanism of COPD [64]. Therefore, we selected TNF-α induced A549 cells as the in vitro inflammatory model. Based on the results of network pharmacology and molecular docking, three bioactive components (luteolin, kaempferol and bergenin) with high response values were selected to perform some verification experiments. Experiments in vitro showed that these three bioactive components could inhibit the increase of IL-6 and MMP9, and alleviate the pathological manifestations of COPD.

## Conclusion

In summary, this study clarified the components of AJH systematically, and explored the molecular mechanisms of AJH against COPD using an integrative strategy for the first time. Totally, 236 components were characterized using UPLC-Orbitrap Fusion MS, of which forty-one key bioactive components screened in AJH may effectively improve the pathologic status of COPD patients by modulating sixty-five corresponding targets primarily involved in inflammation/ metabolism/immune-related pathways. This study provided a comprehensive exploration into the chemical characteristics, pharmacological effects of AJH, and provided a reference for the further study and clinical application of AJH in the treatment of COPD. However, more experiments are needed to further demonstrate the deeper mechanisms, which will be the focus of our research in the future.

## Supporting information

S1 Fig The total ion chromatograms (TICs) of AJH in three batches. (PDF)

S2 Fig The docking results of luteolin, kaempferol and bergenin binding to the five targets. (PDF)

S1 Table The detailed information of components identified in AJH based on UPLC-Orbitrap Fusion MS. (XLSX)

S2 Table The details of network pharmacology results. (XLSX)

S1 File (DOCX)

## Abbreviations

AJH: Ardisiae Japonicae Herba
COPD: chronic obstructive pulmonary disease
UPLC: Orbitrap Fusion MS, Ultra-high performance liquid chromatography-Orbitrap Fusion mass spectrometry
MTT: Thiazolyl Blue Tetrazolium Bromide
FBS: Fetal bovine serum
ELISA: Enzyme-linked immunosorbent assay
PPI: protein-protein interaction
GO: Gene Ontology
KEGG: Kyoto Encyclopedia of Genes and Genomes
Bc: betweenness centrality
Cc: closeness centrality
BP: biological process
MF: molecular function
CC: cellular component
TICs: Total ion chromatograms
t_R_: retention time
AKT1: AKT Serine/Threonine Kinase 1, IL6, Interleukin-6
TNF: Tumor necrosis factor
TP53: Tumor Protein P53
MMP9: Matrix Metalloproteinase 9
