# Supplementary material for: Systematic characterization of the effective constituents and molecular mechanisms of Ardisiae Japonicae Herba using UPLC-Orbitrap Fusion MS and network pharmacology
Source: PLoS One. 2022 Jun 15;17(6):e0269087. doi: 10.1371/journal.pone.0269087 (PMC9200335; doi:10.1371/journal.pone.0269087)
Supplement: S1 Fig — (PDF) [file pone.0269087.s001.pdf]

A1

B1

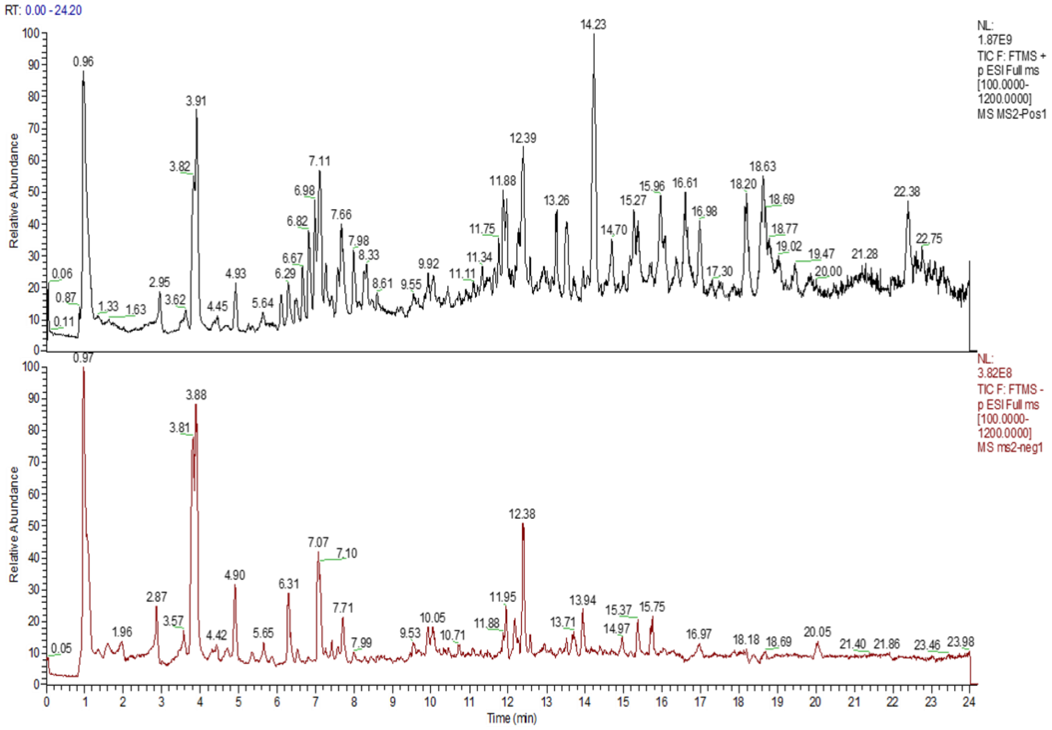
RT: 0.00 - 24.20
Relative Abundance
NL:
1.87E9
TIC F: FTMS + p ESI Full ms [100.0000-1200.0000]
MS MS2-Pos1
NL:
3.82E8
TIC F: FTMS - p ESI Full ms [100.0000-1200.0000]
MS ms2-neg1
Time (min)

A2

B2

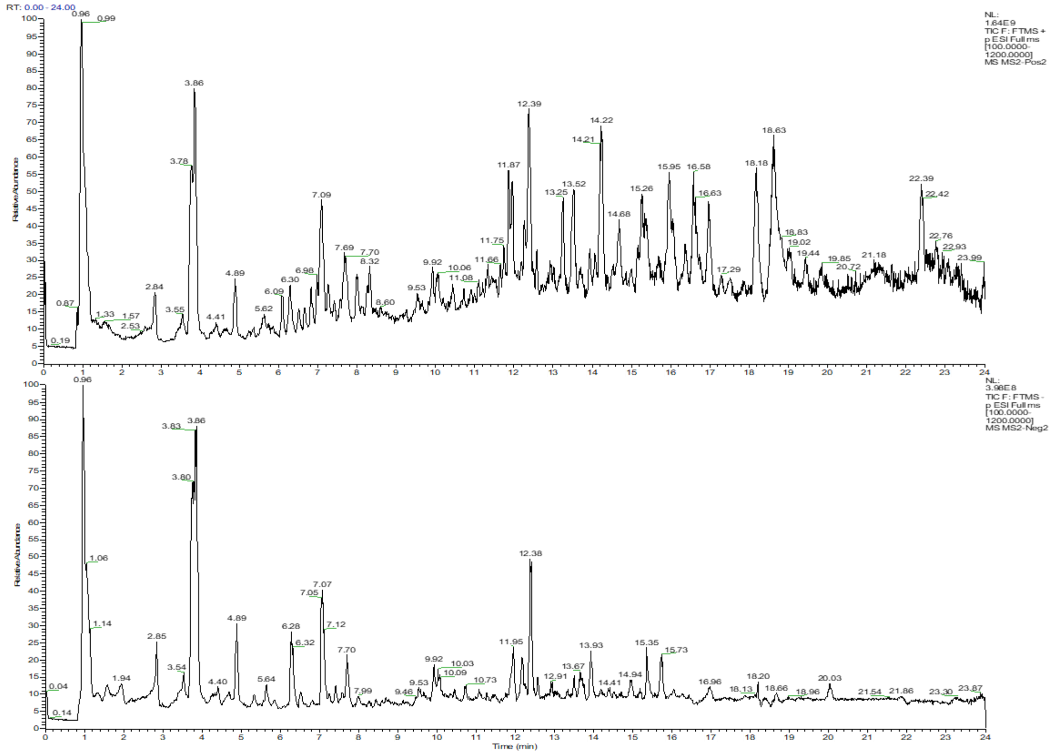
RT: 0.00 - 24.00
NL:
1.64E9
TIC F: FTMS + p ESI Full ms [100.0000-1200.0000]
MS MS2-Pos2
NL:
3.98E8
TIC F: FTMS - p ESI Full ms [100.0000-1200.0000]
MS MS2-Neg2
Time (min)

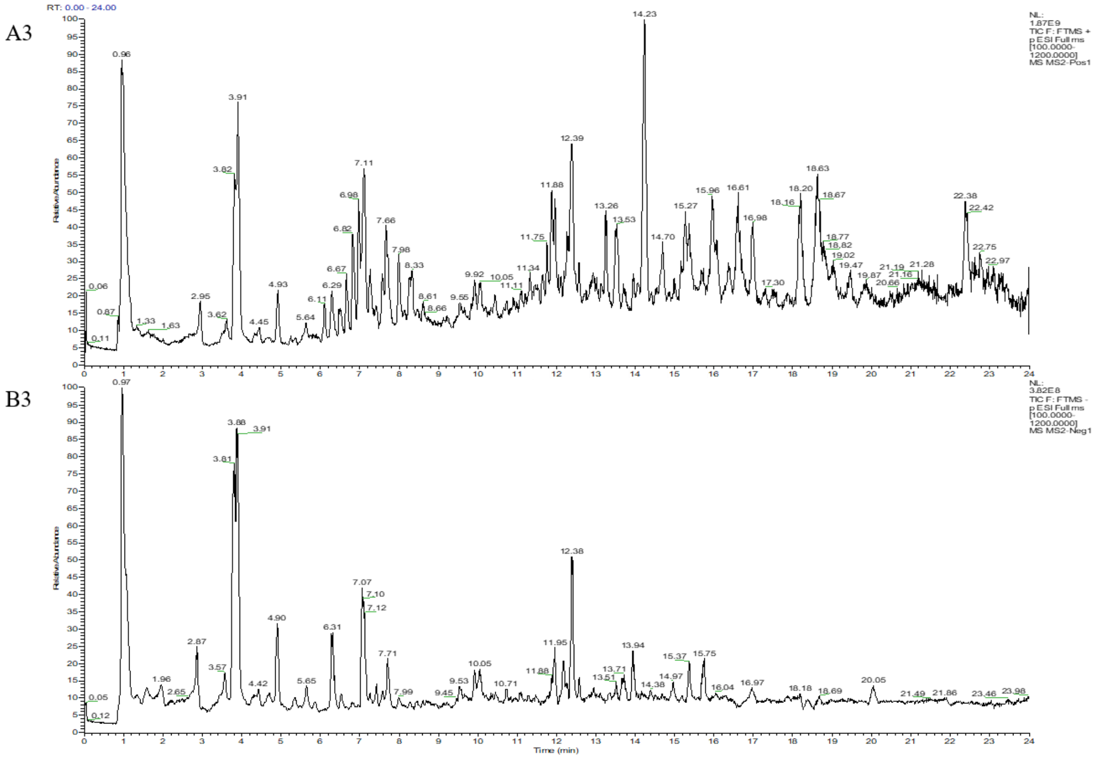

S1 Fig. The total ion chromatograms (TICs) of AJH in three batches (A1-A3. positive ion mode of AJH in three batches, respectively. B1-B3. negative ion mode of AJH in three batches, respectively).
