# Supplementary material for: Systematic characterization of the effective constituents and molecular mechanisms of Ardisiae Japonicae Herba using UPLC-Orbitrap Fusion MS and network pharmacology
Source: PLoS One. 2022 Jun 15;17(6):e0269087. doi: 10.1371/journal.pone.0269087 (PMC9200335; doi:10.1371/journal.pone.0269087)
Supplement: S2 Fig — (PDF) [file pone.0269087.s002.pdf]

(A1)

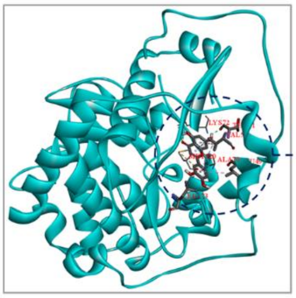

(F1)

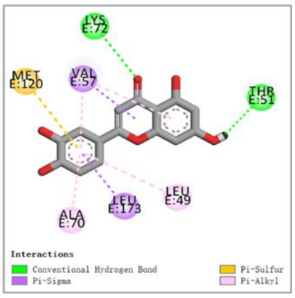

(B1)

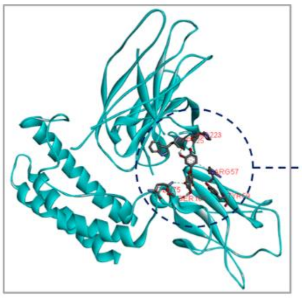

(G1)

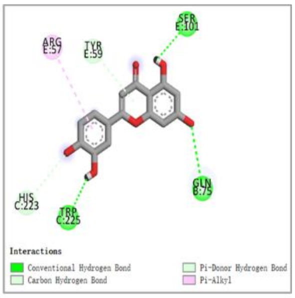

(C1)

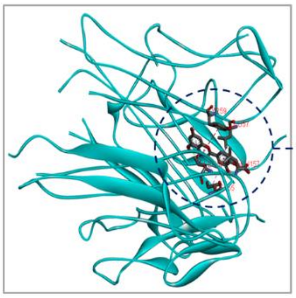

(H1)

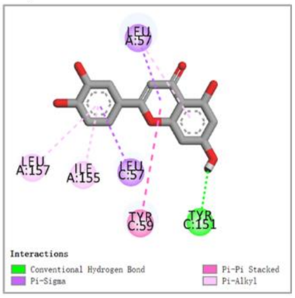

(D1)

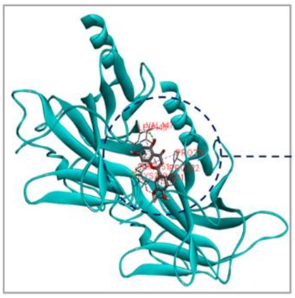

(I1)

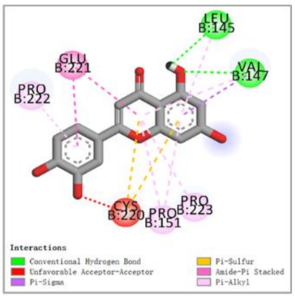

(E1)

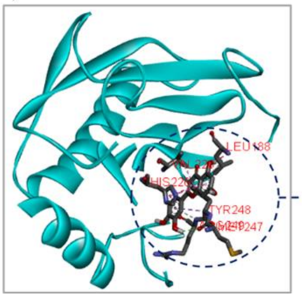

(J1)

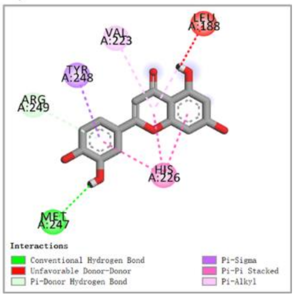

(A2)

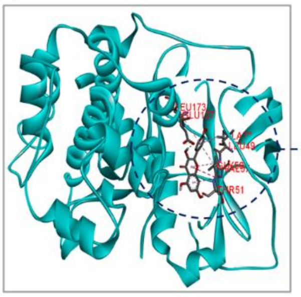

(F2)

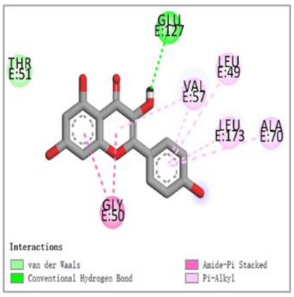

(B2)

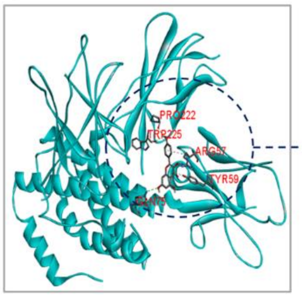

(G2)

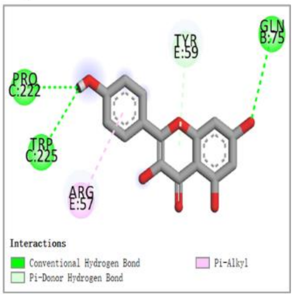

(C2)

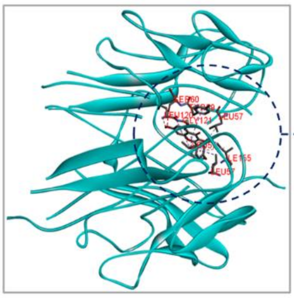

(H2)

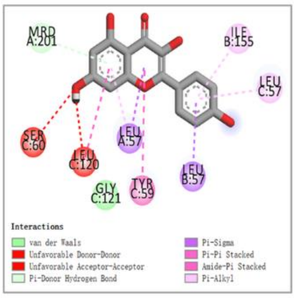

(D2)

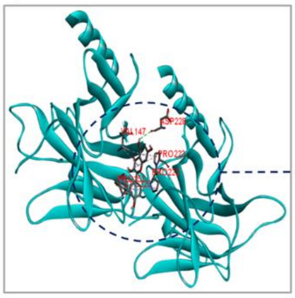

(I2)

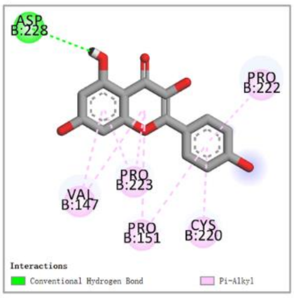

(E2)

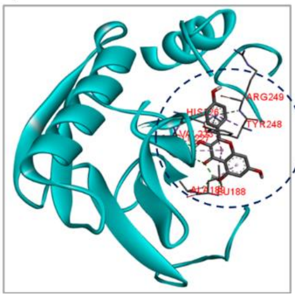

(J2)

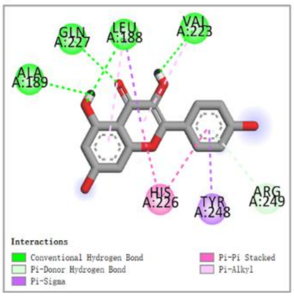

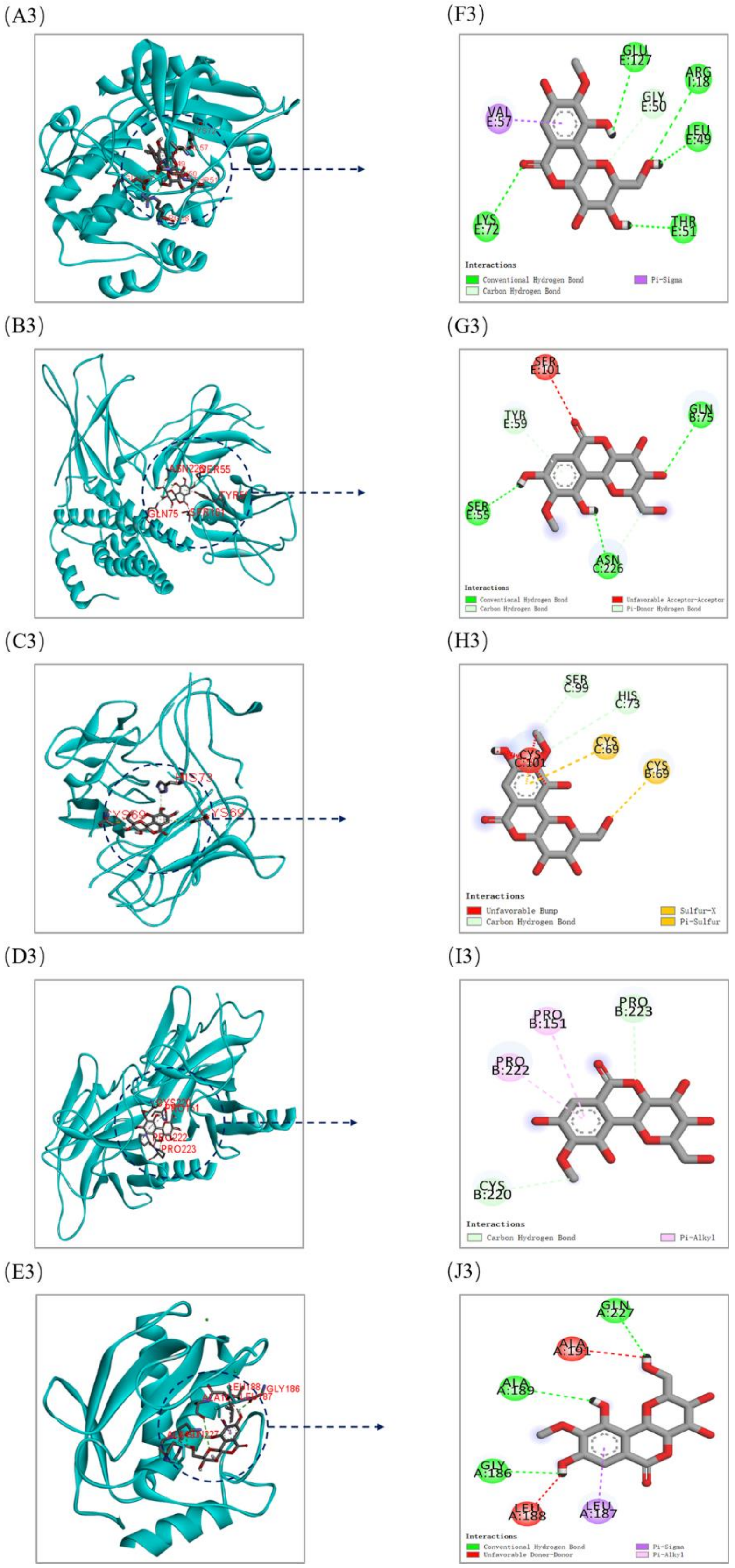

S2 Fig. Docking results of luteolin, kaempferol and bergenin binding to the five targets. (A1-E1) Luteolin docked with AKT1, IL6, TNF, TP53, MMP9, respectively. (F1-J1) The binding interaction between luteolin and AKT1, IL6, TNF, TP53, MMP9 proteins, respectively. (A2-

E2) Kaempferol docked with AKT1, IL6, TNF, TP53, MMP9, respectively. (F2-J2) The binding interaction between kaempferol and AKT1, IL6, TNF, TP53, MMP9 proteins, respectively. (A3-E3) Bergenin docked with AKT1, IL6, TNF, TP53, MMP9, respectively. (F3-J3) The binding interaction between bergenin and AKT1, IL6, TNF, TP53, MMP9 proteins, respectively.
