# Supplementary material for: Systematic characterization of the effective constituents and molecular mechanisms of Ardisiae Japonicae Herba using UPLC-Orbitrap Fusion MS and network pharmacology
Source: PLoS One. 2022 Jun 15;17(6):e0269087. doi: 10.1371/journal.pone.0269087 (PMC9200335; doi:10.1371/journal.pone.0269087)
Supplement: S1 File — (DOCX) [file pone.0269087.s005.docx]

**Systematic characterization of the effective constituents and molecular mechanisms of Ardisiae Japonicae Herba using UPLC-Orbitrap Fusion MS and network pharmacology**

# Methodology

## Qualitative analysis of AJH

### Sample preparation

AJH powder (1.0 g) was accurately weighed and added in a 150 mL round-bottom flask with 100 mL methanol, mixed well, reflux-heated for 1h twice and filtered. Obtained filtrates were combined and dried by centrifugation. The residue was redissolved with methanol (HPLC-grade) and diluted to 10 mL volumetric flask. The solution was filtered using 0.22 μm microporous membrane and stored at 4 °C before analysis.

### Standard solution preparation

Accurately weigh an appropriate amount of each reference standard into a 10 mL volumetric flask, add methanol to dissolve and dilute to the mark, single-component reference stock solutions were obtained and stored at 4 °C before use. Then, proper amounts of reserve solutions were added to a 25 mL volumetric flask, methanol (HPLC-grade) was added to reach scale, the mixed reference solution was obtained. The solutions were filtered through 0.22 μm microporous membranes and stored at 4 °C before use.

### Chromatographic and Mass Spectrometric Conditions

Chemical identification was conducted on an ultra-performance liquid chromatography-orbitrap fusion mass (UPLC-Orbitrap Fusion MS) (Thermo Fisher, Waltham, USA) equipped with an electrospray ionization source (ESI). Thermo Scientific Accucore^TM^C_18_ column (2.1 mm × 100 mm, 2.6 μm) was applied at a constant flow rate of 0.2 mL/min at 30 ℃. The mobile phase was made up of methanol (A) and 0.1% formic acid aqueous solution (B), and the gradient elution was as follows: 0~2 min (93~75% B), 2~10 min (75~20% B), 10~15 min (16~12% B), 15~20 min (12~10% B), 22~24 min (10~0% B). The injection volume was 5 μL.

The acquisition parameters of orbitrap fusion were set as follows: Vaporizer temperature: 275 ℃; Ion Transfer Tube temperature: 300 ℃; Carrier gas (N2); Sheath gas and Aux gas flow were set as 35 arb and 5 arb, respectively; spray voltage were set at 3.5 kV, 2.8 kV (positive and negative ion mode, respectively); collision energy was 35-55 eV. The sample was analyzed in both positive and negative ions Full MS/dd-MS2 modes with the first-level full scan (resolution: 120,000) and the second-level scan (resolution: 60,000), and the mass range was recorded from m/z 100-1200.

### Data processing

The chemical compounds of AJH were collected from existing databases, including the Traditional Chinese Medicine Systems Pharmacology Database and the Analysis Platform (TCMSP, http://lsp.nwu.edu.cn/tcmsp.php), Traditional Chinese medicine integrative database (TCMID, http://47.100.169.139:8000/tcmid/) and SciFinder (https://scifinder.cas.org/) database. Then, a database of AJH ingredients was established. AJH was identified by the optimized UPLC-Orbitrap Fusion MS method. The possible chemical composition (with an error of less than 5 ppm) was determined using Xcalibur based on map data, precise molecular weight and resulting fragment ions. The data was analyzed using Compound Discoverer software to integrate ion peak information, attribution information, ChemSpider, mzCloud and other databases of characteristic fragments integrated with existing chemical composition information reports. The structure of compounds was inferred using the cracking prediction of MassFrontier and its cracking rule.

## Network pharmacology

### Target prediction

The molecular structures of components identified in AJH were downloaded from PubChem database (https://pubchem.ncbi.nlm.nih.gov/) and saved in SDF format. These SDF documents were imported to SwissTargetPrediction database (http://www.swisstargetprediction.ch/) for target prediction. For more accurate prediction, relevant parameters were set (probability ≥ 0.1). In addition, the COPD-related targets were selected from GeneCards database (https://www.genecards.org/) by using “Chronic Obstructive Pulmonary Disease” as the keywords. Then, the overlapping of AJH prediction targets and COPD-related targets was selected as potential targets.

### PPI network construction

The PPI network construction and analysis were performed on Cytoscape 3.7.2 software. Potential targets screened above were added to STRING database. The screening condition used was “Homo sapiens,” and the other parameters were set by default, a protein-protein interaction (PPI) network was built. Cytoscape 3.7.2 was used for topological analysis of PPI network to realize its visualization. Using three topological parameters, betweenness centrality (Bc), closeness centrality (Cc) and degree, a topology analysis of the PPI network was performed to determine hub genes for further analysis.

### Gene Ontology (GO) and Kyoto Encyclopedia of Genes and Genomes (KEGG) enrichment analysis

Potential targets identified above were uploaded into Metascape database (https://metascape.org/gp/index.html) for GO and KEGG analysis to obtain the information of pathways. Bioinformatics Data analysis and Visualization online platform (http://www.bio-informatics.com.cn/) was used to conduct GO and KEGG pathway analysis and visualization. In the process, the organism was set as “Homo sapiens”, and significance level was p ≤ 0.01.

### Construction of component-target-pathway network

To further clarify the relationship among components, targets and pathways, Cytoscape 3.7.2 software was used to build a component-target-pathway network. The core compound nodes were obtained based on the three parameters: degree, Bc and Cc.

### Molecular docking

The affinity between the core compounds and targets was verified by Autodock Vina software The 3D structures of core targets in the first five degrees of AJH in COPD treatment were obtained from the RCSB PDB database (https://www.rcsb.org/). Pymol 1.8 software was used to remove water molecules and separate the primary ligand. After saving, the structure was imported into Autodock Tools 1.5.6 and saved in “pdbqt” format. Chem 3D software was used to download the mol2 files of the top 10 core compounds, and they were imported into Autodock Tools 1.5.6 and saved in pdbqt format. Finally, docking was carried out through Autodock vina 1.1.2. Discovery Studio 4.5 Client was used to visualize docking results and establish a docking interaction model diagram.

## Experimental validation in vitro

### Cell Culture

A549 cells were from Cell Resource Center of Shanghai Academy of Biological Sciences, Chinese Academy of Sciences (Shanghai, China). Cells were cultured in RPMI 1640 complete medium (containing 10% FBS) and kept in an incubator at 37 °C and 5% CO2. When the cells were overgrown, they were digested with 0.25% trypsin-EDTA, collected, subcultured or tested.

### Cell Viability Assay

A549 cells (1 × 10^4^ cells/well) were planted into 96-well plates (n=6), and cultured at 37 °C and 5% CO2 for 24 hours. Then the cells were treated with PBS or different dosages of bergenin (10, 20, 40, 50 μg/mL), luteolin (0.1, 0.2, 0.4, 0.8 μg/mL) and kaempferol (2.5, 5, 10, 20 μg/mL). After incubation for 24 h, added 10 µl MTT (0.5 mg/mL) to each well, and cultured for 4h. Using DMSO (100 µL/well) to replace cell culture. The absorbance was measured at 570 nm using a microplate reader.

### IL-6, MMP9 expression

A549 cells (2×10^6^ cells/plate) were incubated in 6-well plates (n=6) for 24 h, incubated with TNF-α (10ng/mL) for 24 h. Then treated with bergenin (10, 20, 40, 50 μg/mL), luteolin (0.1, 0.2, 0.4, 0.8 μg/mL), and kaempferol (2.5, 5, 10, 20 μg/mL), and incubated at 37 °C with 5% CO2 in a humidified atmosphere for 48 h. Supernatant was collected, centrifuged and stored at -80 ℃ for testing. RIPA Lysis Buffer 300 μL/well was added to lysate cells. Ten minutes later, the lysate was collected, the supernatant was collected by centrifugation, and levels of IL-6, MMP9 in the supernatant were determined according to the instructions of ELISA kits.

### Statistical analysis

IBM SPASS Statistic 26 software was applied for statistical analysis, and data was expressed as mean ± standard deviation (SD). Analysis of variance (ANOVA) was used to analyze the differences between multiple groups. P-values < 0.05 was considered to be statistically significant.
